# Supplementary figures and images for: Overexpression of MdCPK1a gene, a calcium dependent protein kinase in apple, increase tobacco cold tolerance via scavenging ROS accumulation
Source: PLoS One. 2020 Nov 19;15(11):e0242139. doi: 10.1371/journal.pone.0242139 (PMC7676694; doi:10.1371/journal.pone.0242139)

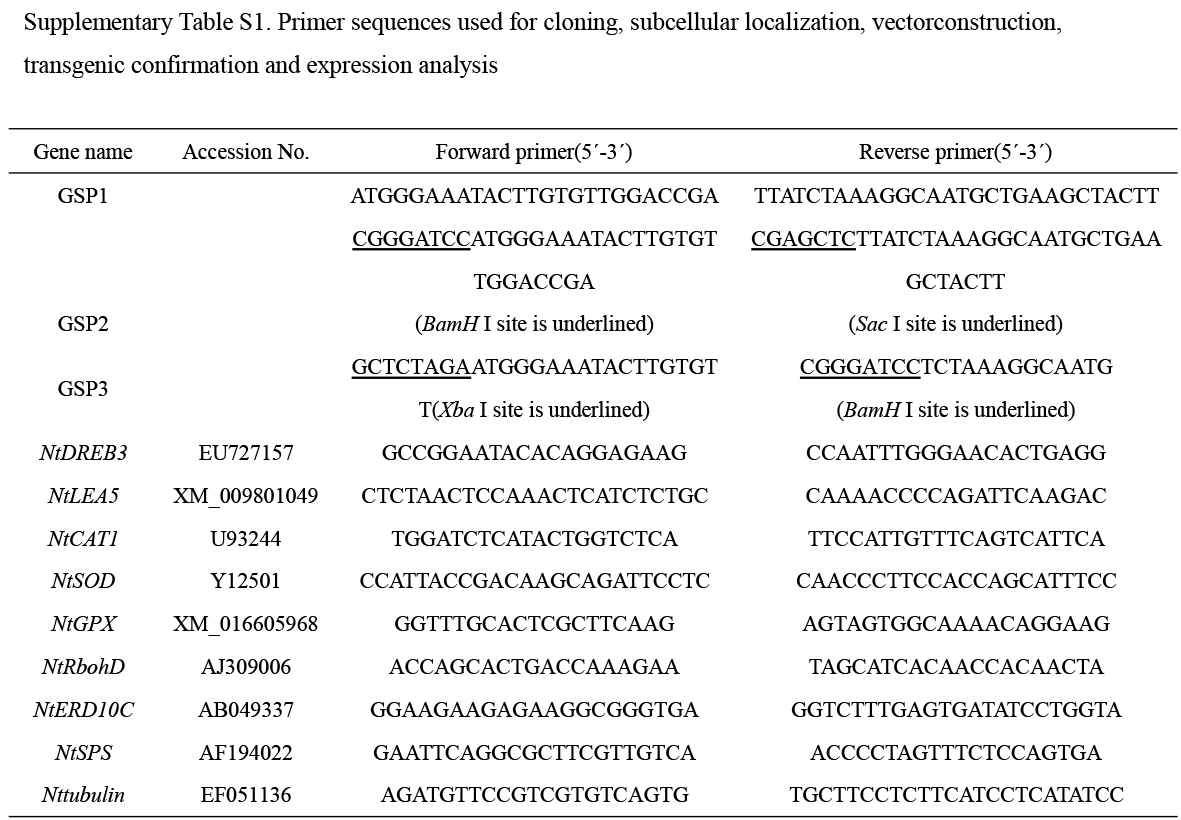

Supplement: S1 Table — (TIF) [file pone.0242139.s001.tif]

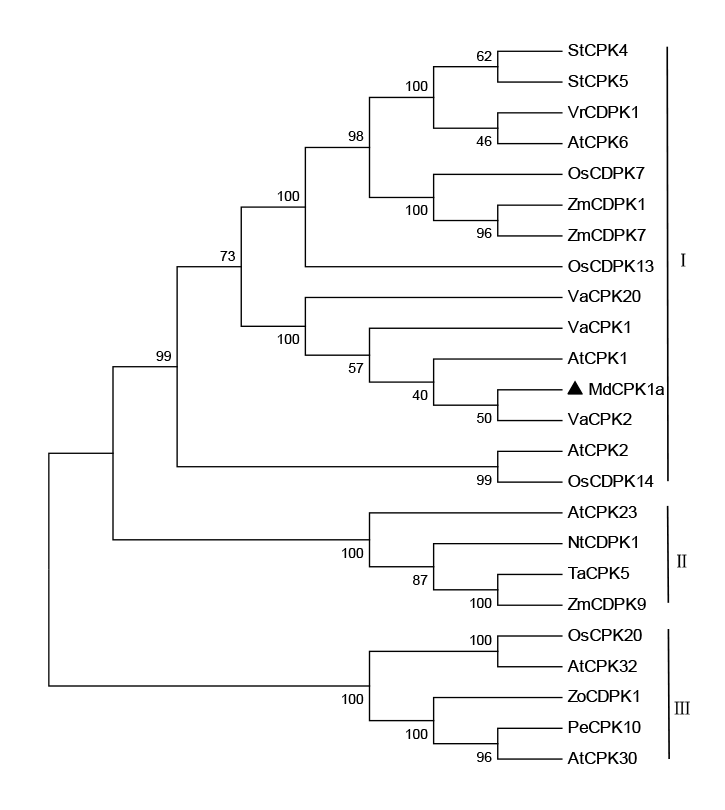

Supplement: S1 Fig — The unrooted tree was generated using MEGA 6.0 program (http://www.megasoftware.net/) by the neighbor-joining method. Bootstrap supports from 500 replicates are indicated at each branch. (TIF) [file pone.0242139.s002.tif]

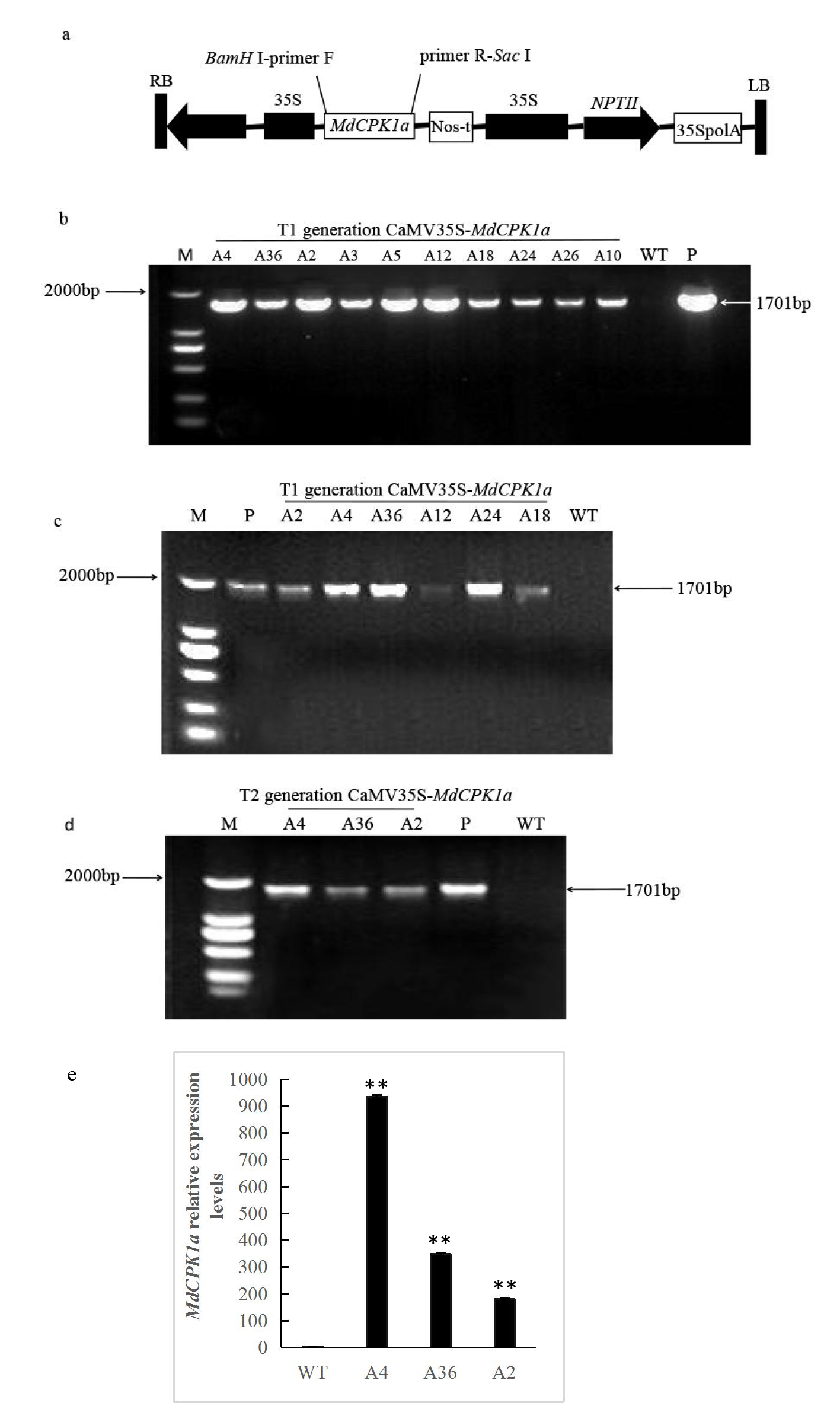

Supplement: S2 Fig — (a) Schematic representations of the vector constructs of pYH455-MdCPK1a. (b) Ten T1 lines of transgenic tobacco were confirmed by PCR with specific primers (PST1). (c) Six T1 lines of transgenic tobacco were confirmed by RT-PCR with specific primers (PST1). (d) Three T2 lines of transgenic tobacco were confirmed by RT-PCR with specific primers. (e) Quantification the expression of MdCPK1a mRNAs in the transgenic tobacco plants performed by real-time RT-PCR. RNA was extracted from the leaves of WT and MdCPK1a-transformed tobacco plant lines (A4, A36, and A2). Transcript abundance was normalized against the Nttubulin gene expression level. Data represent means and standard errors of three replicates. Significant differences between the WT and transgenic plants are indicated by asterisks(*p< 0.05, **p < 0.01). (TIF) [file pone.0242139.s003.tif]

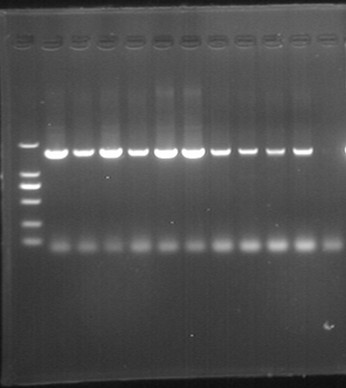

Supplement: S1 Raw images — (ZIP) [file pone.0242139.s004.zip › original underlying images of gel/supplementary figure2b(raw gel)-.jpg]

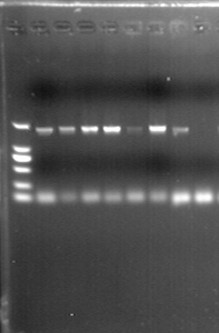

Supplement: S1 Raw images — (ZIP) [file pone.0242139.s004.zip › original underlying images of gel/supplementary figure2c-(raw gel).jpg]

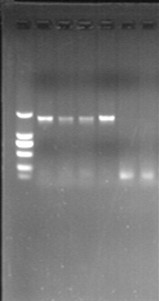

Supplement: S1 Raw images — (ZIP) [file pone.0242139.s004.zip › original underlying images of gel/supplementary figure2d-(raw gel).jpg]
