# Supplementary material for: Overexpression of MdCPK1a gene, a calcium dependent protein kinase in apple, increase tobacco cold tolerance via scavenging ROS accumulation
Source: PLoS One. 2020 Nov 19;15(11):e0242139. doi: 10.1371/journal.pone.0242139 (PMC7676694; doi:10.1371/journal.pone.0242139)

1. Western blot immunoblotted with anti-GFP antibody. The proteins of T,Cm,Cy and Cn fractions were extracted from leaves transciently transformed 35S::GFP


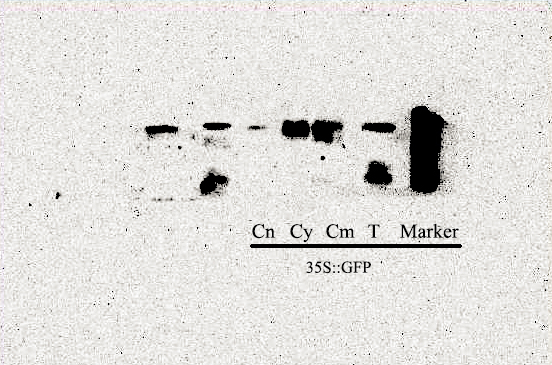


1. Western blot immunoblotted with anti-GFP antibody. The proteins of T,Cm,Cy and Cn fractions were extracted from leaves transciently transformed 35S::MdCPK1a-GFP


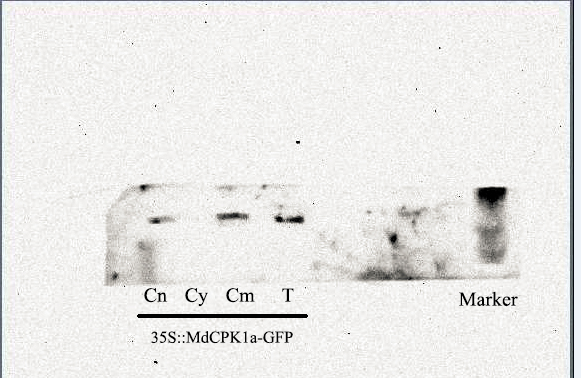

Supplement: S1 Raw images — (ZIP) [file pone.0242139.s004.zip › original underlying images of gel/WB raw gel.docx]
