# Supplementary material for: Overexpression of MdCPK1a gene, a calcium dependent protein kinase in apple, increase tobacco cold tolerance via scavenging ROS accumulation
Source: PLoS One. 2020 Nov 19;15(11):e0242139. doi: 10.1371/journal.pone.0242139 (PMC7676694; doi:10.1371/journal.pone.0242139)
Supplement: Attachment — Submitted filename: Response to reviewers.docx [file pone.0242139.s005.docx]

**Dear editor,**

We have received the comments on our manuscript entitled “Overexpression of *MdCPK1a* gene, a calcium dependent protein kinase in apple, increase tobacco cold tolerance via scavenging ROS accumulation” by Dong et al. We thank very much for the valuable suggestions and good comments of reviewers. We have carefully considered the comments and responded point by point to each reviewer’s comments as listed below. We also submitted the revised manuscript make-up copy as well as clean copy.

Hope these responses and the revision will make it more acceptable for publication.

Your Sincerely,

Dong, H

**Response to the editors:**

1. *When submitting your revision, we need you to address these additional requirements. Please ensure that your manuscript meets PLOS ONE's style requirements, including those for file naming.*

**-------We changed the revised manuscript style according to PLOS ONE’s style requirements.**

2. *For reproducibility reasons we would recommend that you amend your methods section to include the source and/or deposition numbers of all the plants and seeds used in your study. We would also recommend providing some minimal details regarding the protocols references in section "Physiological measurements and histochemical staining protocol details" to allow researchers that may not have access to these references to reproduce your findings.*

**--------In the revised manuscript, we provided more details in the section “Physiological measurements and histochemical staining”**

3. *PLOS ONE now requires that authors provide the original uncropped and unadjusted images underlying all blot or gel results reported in a submission’s figures or Supporting Information files.*

*In your cover letter, please note whether your blot/gel image data are in Supporting Information or posted at a public data repository, provide the repository URL if relevant, and provide specific details as to which raw blot/gel images, if any, are not available. Email us at plosone@plos.org if you have any questions.*

**-------- We have provided raw blot/gel images in supporting information.**

4. *Thank you for stating in your Funding Statement: [This work was supported by the National Natural Science Foundation of China (31872076, 31560551).].*

** Please provide an amended statement that declares *all* the funding or sources of support (whether external or internal to your organization) received during this study, as detailed online in our guide for authors at* [*http://journals.plos.org/plosone/s/submit-now*](http://journals.plos.org/plosone/s/submit-now)*.  Please also include the statement “There was no additional external funding received for this study.” in your updated Funding Statement. Please include your amended Funding Statement within your cover letter. We will change the online submission form on your behalf.*

**------- We have provided all the funding. According the instruction, we included “There was no additional external funding receives for this study” in our updated funding statement.**

Reviewer #1:

1. *Authors indicated the nuclear and cytosolic localization of MdCPK1a by GFP fusion protein.* ***But,*** ***nuclear localization should be confirmed by*** ***DAPI staining.***
   *---------* Thank you for the reviewer’s suggestion! There is no doubt that the nuclear localization confirmed by DAPI staining can further guarantee the credibility of our result. We repeated the subcellular localization experiment for several times and the same results were obtained. We regretted that we did not take the photos of the DAPI staining. However, DAPI staining is not necessary for confirming nuclear localization. For example, a recent study revealed that some alternative splicing (AS) forms of OsCPK17 located in cytoplasm and nucleus. In this report, they constructed GFP-fused OsCPK17 AS isoform vectors (*GFP-OsCPK17.1/2, GFP-OsCPK17.3, GFP-OsCPK17.4,* and *GFP-OsCPK17.5* or otherwise free GFP as a control). They used  [particle bombardment](https://www.sciencedirect.com/topics/biochemistry-genetics-and-molecular-biology/biolistic-transformation) to identify OsCPK17 AS isoform [subcellular localization](https://www.sciencedirect.com/topics/biochemistry-genetics-and-molecular-biology/subcellular-localization) in onion epidermal cells, and showed the subcellullar localization results in the Figure 3, in which they presented fluorescence projection, differential interference contrast (DIC) and merge images, but lack of DAPI staining or other nuclear markers (Almadanim et al, 2018). The similar case can also be found in the article Li et al.(2009), Plant Cell,21(2):429-441, including its correction at [January 01,](http://www.plantcell.org/content/30/1/249) (2018). Plant Physiology, 177(3):1339-1341. In other case, to confirm the localization of some proteins on small organelles, such as on Golgi , Microsome, et al. It requires the organelle-markers (Almadanim et al, 2018).

Reference:

(1) Almadanim, M. C., Goncalves, N. M., Rosa, M. T., Alexandre, B. M., Cordeiro, A. M., Rodrigues, M., ... & Abreu, I. A. (2018). The rice cold-responsive calcium-dependent protein kinase OsCPK17 is regulated by alternative splicing and post-translational modifications. *Biochimica et Biophysica Acta (BBA)-Molecular Cell Research*, *1865*(2), 231-246.
(2) Li, S., Lauri, A., Ziemann, M., Busch, A., Bhave, M., & Zachgo, S. (2009). Nuclear activity of ROXY1, a glutaredoxin interacting with TGA factors, is required for petal development in *Arabidopsis thaliana*. The Plant Cell, 21(2), 429-441.
(3) Plant Physiology Jul 2018, 177 (3) 1339-1341; DOI: 10.1104/pp.18.0066
2. *In the picture taken from the top (“a” on the left panel) in Fig.3, A2 plants seem to be much larger than WT plants. However, shoot and root dry weight of A2 are comparable with that of WT plants.* ***This discrepancy between picture and graph should be explained.***

------Thank for your careful reading. After salt stress, A2 plants seem to be much larger than WT plants,while the shoot and dry weight of A2 are comparable with that of WT plants. It may be that WT plants displayed yellowing and wilted leaves under salt stress, while the leaves of transgenic plants were green and leaves fully extended, so 3 transgenic lines all seem to be larger than WT plants from the overall looking, but for the individual plants, the plant high and root size between WT and A2 plants have no significant difference, which can be seen in Fig.3(“a” on the right panel).

*3. In this study, the results presented in Figure 7 are essential to support the main conclusions. Authors therefore should quantify amount of ROS with more accurate way.* ***Especially, the ways to quantify H_2_O_2_ by*** ***fluorescent dye or kit have been established.***

------ We agreed the reviewer’s comment. If we measured the content of ROS, it can provide the direct evidence, but now, for lack of the growing transgenic tobacco plants, it is difficult for us to supplement the experiment in the short time. Actually, based on the histochemical staining to determine the level of the accumulation of H_2_O_2_ and O_2_^-^ has been used in many papers, such as Shen et al (2016), Trevisan et al.(2019) and Sahoo et al.(2019). For better describing the degree of staining, we supplement the column diagram of the percentage of staining area according to the statistic results of five staining leaves for each treatment in Figure 7 of the revised manuscript, which could quantify the amount of ROS indirectly.

Figure 7

Reference:

Shen, L., Yang, S., Yang, T., Liang, J., Cheng, W., Wen, J., ... & Shi, W. (2016). CaCDPK15 positively regulates pepper responses to Ralstonia solanacearum inoculation and forms a positive-feedback loop with CaWRKY40 to amplify defense signaling. *Scientific reports*, *6*, 22439.

Trevisan, Sara, et al. Nitrate affects transcriptional regulation of UPBEAT1 and ROS localisation in roots of *Zea mays* L.  *Physiologia plantarum* 166.3 (2019): 794-811.

Sahoo, S., Saha, B., Awasthi, J. P., Omisun, T., Borgohain, P., Hussain, S., ... & Panda, S. K. (2019). Physiological introspection into differential drought tolerance in rice cultivars of North East India. Acta Physiologiae Plantarum, 41(4), 53.

4. *Although the stress response phenotypes of the transgenic plants are shown in this study, the effects of MdCPK1a expression on growth of transgenic plants under non-stressed conditions are not clearly shown. The growth phenotypes of* *transgenic plants need to be analyzed and presented.*

--------The growth phenotypes of transgenic plants has been presented in this paper actually. The Fig 6a shows the phenotype of WT, A4, A36 and A2 at the normal growth temperature for 10 days on MS medium after germinated. It showed that the root length of transgenic plants A4 and A36 is longer, but only of A4 is significant longer than that of WT. In the panel of “before drought” of Figure 4 , the growth of transgenic plants under the normal conditions are also shown. It shows that the aerial part of WT plants is little higher than that of the transgenic plants under normal condition. We also speculated that MdCPK1a might participate in the regulation of plant development.

Thus，in the discussion of revised manuscript, in line 449-454, we added the discussion as below: “Additionally, the root length of transgenic plants A4 and A36 is longer than that of WT when the seedlings of them cultured at 25℃ for 10 d on MS medium( Fig 6a) . The aerial part of WT plants is a little higher than that of the transgenic plants under normal condition (Fig 4). We speculated that MdCPK1a might also participate in the regulation of plant development.”

**Reviewer #2:**
Major comment:
*1. The 3 transgenic lines should behave similarly to link the phenotype to the expression of MdCPK1a, or at least follow the overexpression level. Yet, it is often not the case. For heat stress, the higher MdCPK1a is expressed, the lower the tolerance is induced (Fig 5) and the authors claim in the abstract l.16 that “Ectopic expression of MdCPK1a in Nicotiana benthamiana increased its salt, heat and cold resistance” while in the discussion, they state l.303-304: “ectopic expression of MdCPK1a improved tobacco cold tolerance and also exhibit slightly increased salt tolerance, but no obvious improvement of heat and drought tolerance”. For cold, lines A4 and A36 already appear much bigger than WT in control conditions when grown on plates (Fig 6a), which questions whether the increased tolerance to cold is specific or just a consequence of initial bigger size. Moreover, the analyzed molecular parameters, i.e. enzyme activity and gene expression, are not consistent in the 3 transgenic lines, which makes it difficult to link those responses to MdCPK1a overexpression.*

--------We apologized for confusing the reader because of our carelessness. Ectopic expression of *MdCPK1a* improved tobacco cold and salt tolerance, but no obvious improvement of heat and drought tolerance.The description in the summary is incorrect and has been modified.

AS for the comment “whether the increased tolerance to cold is specific or just a consequence of initial bigger size?” CDPK regulating development in plant has been reported in some studies ( see review by Singh et al. 2017 ). In our study, we also supposed that *MdCPK1a* might involve in plant development (Fig 6a). We added the discussion in L449-L454. On the other hand, we don’t think that the bigger size of transgenic plants will influence on the cold resistance ability in this experiment. We think the cold stress related parameters, such as the content of MDA，the electrolyte leakage，the enzyme activities, et al. do not change with the plant size.

In our research, it was no doubt that the stress related enzyme activities and the stress related gene expression of the transgenic plants were higher that those of control plant. The transgenic tobacco plants are more resistant to cold and salt stresses as result of overexpression of *MdCPK1a*. Why are the stress related enzyme activities and gene expression not consistent with the gene expression of MdCPK1a ? We explained that the posttranscriptional or posttranslational modification of MdCPK1a might exist in *MdCPK1a -OX* plants, which might be influenced by the insertion locus of MdCPK1a.

**Reference:**

Singh, A., Sagar, S., & Biswas, D. K. (2017). Calcium dependent protein kinase, a versatile player in plant stress management and development. Critical reviews in plant sciences, 36(5-6), 336-352.

*In Fig 2, the fluorescent signal of MdCPK1a-GFP being very weak, it is difficult to conclude that MdCPK1a localizes to* *plasma membrane and not cytosol. The authors should improve the quality of the pictures and check by western-blot that the signal corresponds to the fusion protein and not to GFP alone.*

**-------** Indeed, the fluorescent signal of MdCPK1a-GFP is weak on plasma membrane. We also believe it will make our conclusion more convincing if we conducted western-blot. The similar comment was given on the subcelluar localization by Reviewer #1. Please refer to our response to the comment to Reviewer #1. According to the reviewer’s suggestion, we improved the quality of the image. It became more clearly.

3. *The literature is not always relevant.*

------Thank you very much for careful reading! According the reviewer’s suggestion, we corrected the references accordingly.

*For example, l. 33 ref 52 is more relevant than ref 1.*

------We corrected it.

L. *45, ref 6 and 10 are not relevant here.*

------We deleted the two reference.

*L. 263, ref 51 doesn’t deal with AtCPK1. Instead, the authors should cite ref 48 and* *Gao et al Plos Pathogen 2013 vol 9: e1003127. And Ref 52 is not relevant there.*

-------We corrected it.

*L. 298, the authors should include* *OsCPK24 (Liu et al 2018 Journal of Integrative Plant Biology vol 2 p.173-188).*

-------We added *OsCPK24* in the manuscript, and cited the reference by Liu et al (2018).

*L. 326, ref 74 is not relevant. Instead, the authors should cite Boudsocq et al Nature 2010 vol 464 p. 418-22; Dubiella et al, PNAS 2013 vol 110 p. 8744-8749; Gao et al Plos Pathogen 2013; Kadota et al Molecular Cell 2014 vol 54, p. 43-55.*

-------We corrected it.

**Minor comments:**
*1. In Fig 3a, on the left panel, line A2 seems to be as tolerant as lines A4 and A36, which is different in the right panel. Moreover, the 2 panels are not well explained in the legend. The statistics are missing in fig 3b*.

-------The comment was also given by the Reviewer #1. Please refer to our response above. Thank you for your careful reviewing! The shoot dry weight of A4 and A36 (except A2) was significantly higher than that of WT; The root dry weight of A4 (except A36 and A2)was significantly higher than that of WT. We added the statistics on the Fig.3b.

*2. In Fig 7, the ROS should be quantified.*

-------We responded the same comment to the Reviewer #1 on the above.

*3. the stress protocols are not similar in methods and results. They should be clarified*.

-------We read carefully and already kept the stress protocol of methods and results consistent.

*4. the English should be improved*.

------- We polished the English.

*5. Figure legends are inverted in Fig 6 between panels c, d, e and f l. 403-407; sup fig S1 and S2 are inverted l.427, 437*.

------- Thank you so much for your careful reading! We corrected it .
