# Supplementary material for: Overexpression of MdCPK1a gene, a calcium dependent protein kinase in apple, increase tobacco cold tolerance via scavenging ROS accumulation
Source: PLoS One. 2020 Nov 19;15(11):e0242139. doi: 10.1371/journal.pone.0242139 (PMC7676694; doi:10.1371/journal.pone.0242139)
Supplement: Attachment — Submitted filename: response to the reviewers.doc [file pone.0242139.s006.doc]

Thank you for the two reviewers and the editors

We are grateful to your critical reading. Your comments are invaluable for the manuscript conforming to the requirement for publishing. The comments and responses were listed as follows. We hope the revised manuscript will meet the requirements for publication on Plos One.

Reviewer #1: Basically, authors properly answered the comments by explaining the detail. However, I found another point to be modified. Fig.4 might be important pictures that also show difference in growth even under normal condition. Scale bars should be indicated to further provide information of plant size.

**Reply：**Thank you for the reviewer’s suggestion！We have indicated the scale bars in the figure 4 and the legends. Bars =10 cm.

Reviewer #2: The authors answered part of the initial comments but some points still need clarification.

Major comments:

1.The part on heat tolerance is confusing since the authors conclude l. 434-436 that “ectopic expression of MdCPK1a exhibit…no obvious improvement of heat and drought tolerance” while in the results, they state l.310-311 “the survival rates of…A36 (24%) and A2 (98%) were remarkably higher than WT”. Indeed, the thermotolerance is inversely correlated with MdCPK1 expression level in the transgenics, which makes it difficult to conclude without any additional investigation. Thus, the whole part on heat stress tolerance should be deleted from this manuscript.

**Reply:** Thank you for the reviewer’s suggestion. We deleted the whole part of heat stress in the manuscript.

2. The picture of *MdCPK1* localization has been more contrasted. However, the membrane localization is still not demonstrated. Indeed, some cytoplasmic strands are visible and *MdCPK1* could be located in cytosol and/or plasma membrane. This experiment is important to correlate with the acylation prediction of *MdCPK1*. Either the authors balance their statement, or they provide additional data such as co-localization with known plasma membrane protein, or western-blot of proteins extracted in the presence/absence of triton X100 which will extract total proteins including the membrane ones (presence) or only soluble ones (absence). Moreover, a western-blot will additionally prove that the fluorescence observed is due to *MdCPK1*-GFP fusion and not GFP alone. Finally, “plasma membrane” should be deleted l.265-266 since free GFP doesn’t go to membrane. It only diffuses to cytosol and nucleus.

**Reply:** Thank you for your suggestion. We conducted the supplementary experiment of Western Blot to further confirm the subcellular localization of MdCPK1 according to the reviewer’ suggestion.

The Western blot assay showed that MdCPK1a-GFP was exclusively detected in the cell membrane and nucleus fractions, but not in the cytosolic by immunoblotting with anti-GFP antibody (Figure 2c). The results indicated that MdCPK1a protein was localized to the nucleus and cell membrane. The result was added in the revised manuscript and Figure 2 was modified also accordingly..

Minor comments:

1. In Fig3, the discrepancy between pictures (a, left panel) and graphs (b, left panel) is surprising. Maybe measuring fresh weight would have been more relevant here. Nevertheless, the authors could comment on that: it suggests that under salt stress, MdCPK1 transgenics retain water better than WT.

**Reply:** we agreed with the reviewer’s comments that fresh weight would have been more relevant here. However, we believed that dry weight might more accurately reflect difference of the growth and development of lines. A transgenic line will increase more biomass and accumulate more dry matter if it has higher adaptability under certain condition, accordingly, the dry weight of the plant is higher than the others. We don’t think the picture a(left) and graph of b(left) is discrepant.

2. In Fig7, the authors added quantification of the ROS staining. The corresponding legend is missing l.358-362, the panels c and d should be cited in the results l.352, and the way they quantified the staining should be explained in the methods.

**Reply:** We apologized for our carelessness. The way of quantification for the staining have been added in the methods. The corresponding legend has been added for the Fig7 (in the revised manuscript, it was renamed Fig.6) and the panels c and d were cited in the results.

3. In the discussion l.447-448, the authors state that “the aerial part of WT plants is little higher than that of transgenics under normal conditions”. However, the picture in Fig4 shows that WT plants are actually smaller than the transgenics in control conditions. This should be corrected.

**Reply:** We apologized for our carelessness, we have corrected the mistake.

4. The stress protocols are still different in methods and figures. In results, salt stress (l.285) and drought (l.303) are performed for 25d on 4-week-old plants while in the methods, 4-week-old plants are further grown for 14 d before applying stress, ie 6-week-old before stress (l.169-170). Also correct accordingly if needed the legend l.305-307.

**Reply:** Thank you for the reviewer’s careful reading. We corrected the mistyping. The transgenic lines were all treated at 6 weeks old.

5. L.86, *Ralstonia solanac* should be *Ralstonia solanacearum.* L.402, predicated should be predicted.

**Reply:** Thank so much for your comments! we have corrected the error.

6. English still needs some improvement, especially l.89-91, l.113-115, l.178-180.

**Reply: Thank you!** We proofread the whole manuscript and improved the three sentences as follows:

L89-91: Conversely, some *CDPKs* play negative regulators of stress response for  transgenic plants overexpressed them show more sensitive to abiotic/biotic stresses.

L113-115: The fourth and fifth young leaves were taken from the annual branches of the *Malus domestica* cv. ‘Jonathan’growing in the greenhouse. Total RNA was extracted by using CTAB method.

L.178-180: Leaf samples (0.5 g) were homogenized in 2 mL 20% trichloroacetic acid with the aid of some sand, and then the homogenate was centrifuged at 16,000 g for 20 min at 4 °C. The supernatant (1 mL) was mixed with equal volume of 0.5% (w/v) TBA.

7. L.251, the sentence should be rephrased by “the selected CDPK proteins were clustered into three subgroups” because the authors just didn’t include CDPKs from subgroup IV. L.252, ZmCDPK1 should be “ZmCPK1”.

**Reply:** Thank you for your suggestion! We have rephrased the sentence in the revised manuscript.

8. In FigS2, the qRT-PCR (panel e) is missing.

**Reply**: We added the qPCR (panel e) in the new FigS2.

9. Fig6 has been reorganized but the labels are now wrong: a and b are inverted, c and d, and e and f, as well. Moreover, the genotype labelling is missing in panel b (which should be “a” with MS and MS+4°C).

**Reply:** Thank you! We have reorganized the Fig5 and re-labelled (the original figure 6 was revised as figure 5 in the revised manuscript).

10. L.375-378, the sentence should be modified because *NtSPS* and *NtLEA5* are already strongly induced in MdCPK1 transgenics in normal conditions.

**Reply:** we have modified the sentence.

The mRNA levels of cold-responsive genes except *NtERD10C* were higher in the transgenic tobacco than those of WT plants under normal and cold stress condition (Fig 8). The expression of *NtERD10C* in the transgenic tobacco was similar with that in WT under normal condition, but higher under cold stress.
